# Resistance Gene-Directed Genome Mining of 50 *Aspergillus* Species

**DOI:** 10.1128/mSystems.00085-19

**Published:** 2019-05-14

**Authors:** Inge Kjærbølling, Tammi Vesth, Mikael R. Andersen

**Affiliations:** a Department of Biotechnology and Biomedicine, Technical University of Denmark, Kongens Lyngby, Denmark; Genome Institute of Singapore

**Keywords:** *Aspergillus*, comparative genomics, genome mining, bioactive compounds, fungal, genomes, natural products, resistance, secondary metabolism

## Abstract

Fungal secondary metabolites are a rich source of valuable natural products, and genome sequencing has revealed a proliferation of predicted biosynthetic gene clusters in the genomes. However, it is currently an unfeasible task to characterize all biosynthetic gene clusters and to identify possible uses of the compounds. Therefore, a rational approach is needed to identify a short list of gene clusters responsible for producing valuable compounds. To this end, several bioactive clusters include a resistance gene, which is a paralog of the target gene inhibited by the compound. This mechanism can be used to identify these clusters. We have developed the FRIGG (fungal resistance gene-directed genome mining) pipeline for identifying this type of biosynthetic gene cluster based on homology patterns of the cluster genes. In this work, the FRIGG pipeline was run using 51 *Aspergillus* and *Penicillium* genomes, identifying 72 unique families of putative resistance genes. The pipeline also identified the previously characterized resistance gene *inpE* from the fellutamide B cluster, thereby validating the approach. We have successfully developed an approach to identify putative valuable bioactive clusters based on a specific resistance mechanism. This approach will be highly useful as an ever-increasing amount of genomic data becomes available; the art of identifying and selecting the right clusters producing novel valuable compounds will only become more crucial.

**IMPORTANCE** Species belonging to the *Aspergillus* genus are known to produce a large number of secondary metabolites; some of these compounds are used as pharmaceuticals, such as penicillin, cyclosporine, and statin. With whole-genome sequencing, it became apparent that the genetic potential for secondary metabolite production is much larger than expected. As an increasing number of species are whole-genome sequenced, thousands of secondary metabolite genes are predicted, and the question of how to selectively identify novel bioactive compounds from this information arises. To address this question, we have created a pipeline to predict genes involved in the production of bioactive compounds based on a resistance gene hypothesis approach.

## Abstract (precis)

Species belonging to the *Aspergillus* genus are known to produce a large number of secondary metabolites; some of these compounds are used as pharmaceuticals, such as penicillin, cyclosporine, and statin. With whole-genome sequencing, it became apparent that the genetic potential for secondary metabolite production is much larger than expected. As an increasing number of species are whole-genome sequenced, thousands of secondary metabolite genes are predicted, and the question of how to selectively identify novel bioactive compounds from this information arises. To address this question, we have created a pipeline to predict genes involved in the production of bioactive compounds based on a resistance gene hypothesis approach.

## INTRODUCTION

Fungal secondary metabolites are a rich source of bioactive compounds, including important pharmaceuticals such as penicillin, cyclosporine, and statins (1). When the first fungal genomes were sequenced, it became clear that the genomes harbor a higher number of predicted secondary metabolite gene clusters than the number of characterized secondary metabolites, thus revealing a much larger potential (1–4). The number of sequenced fungal genomes is ever increasing, mainly due to large sequencing efforts such as the 1000 Fungal Genomes Project (http://1000.fungalgenomes.org/home/) and the 300 *Aspergillus* genome project (5, 6), and, with them, the number of new biosynthetic gene clusters (clusters).

Despite progress in molecular tools and methods for characterization of such clusters, it is still a time-consuming task, making it unfeasible to investigate all predicted clusters. Therefore, only a small fraction of the clusters is characterized and investigated experimentally. With the plethora of clusters and the aim of discovering novel bioactive compounds useful as drugs, the following question emerges: How do we directly select the most interesting clusters producing potential fungicides, anticancer drugs, and antimicrobial compounds? To meet this need, we have created the “fungal resistance gene-directed genome mining” (FRIGG) pipeline to identify clusters producing likely bioactive compounds. Appropriately for a predictive algorithm, Frigg is the Norse goddess of foresight and wisdom.

**FIG 1:**
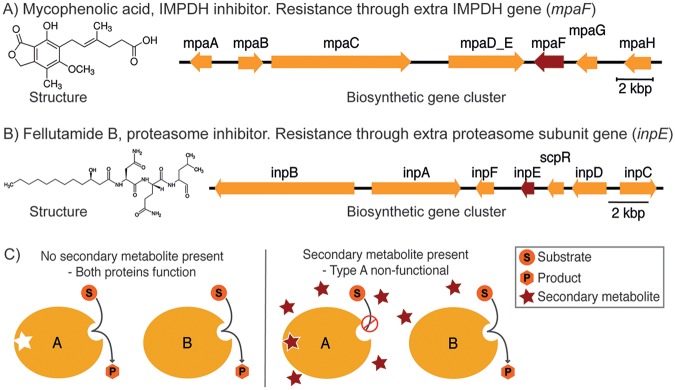
Resistance mechanism. (A) Mycophenolic acid chemical structure and biosynthetic cluster with the known resistance gene *mpaF* (highlighted in red), which is an IMP dehydrogenase (IMPDH) inhibitor. (B) Chemical structure of fellutamide B and overview of the biosynthetic cluster, including the resistance gene *inpE* (highlighted in red), which is a proteasome inhibitor. (C) Illustration of the resistance mechanism used by some toxin producers. The secondary metabolite is a toxin which inhibits an essential enzyme, the target of the compound. Within the cluster responsible for producing the toxin, a copy of the target gene is found; this version is still functioning despite the compound’s presence and hence makes the organism self-resistant.

Many bioactive compounds are toxic compounds that also impair the organisms that synthesize them by inhibiting essential functions; therefore, a self-resistance mechanism is needed in order for the organism to survive (7–9). One known self-resistance mechanism is the duplication of the target gene, where the duplicate is resistant to the compound. This second resistant version is most often located as part of the biosynthetic gene cluster producing the toxic compound. This mechanism has been seen in several bacterial instances, such as novobiocin (10) and pentalenolactone (11, 12), and more recently in fungi. Mycophenolic acid (MPA) is produced by Penicillium brevicompactum, where it inhibits IMP dehydrogenase (IMPDH), the rate-limiting step in guanine synthesis. The biosynthetic cluster of MPA revealed an additional copy of IMPDH (Fig. 1A), which is insensitive to MPA, thus conferring resistance (13–15). Another example is fellutamide B, a proteasome inhibitor produced by Aspergillus nidulans. Within the biosynthetic gene cluster, a gene (*inpE*) encodes a proteasome subunit (Fig. 1B), and it was shown that this gene confers resistance (16).

An illustration of the general mechanism can be seen in Fig. 1C: two versions of an enzyme are present. One version (the target) is affected by the secondary metabolite, whereas the other version (the resistance gene) is not inhibited by the secondary metabolite. Even though only a few examples of this mechanism have been verified in filamentous fungi so far (13, 16–18), it is possible that this resistance mechanism is more widely distributed. We thus developed the FRIGG pipeline for identifying putative bioactive clusters with resistance genes. The aim of the pipeline is to identify bioactive clusters in a targeted manner, thus providing a way of selecting the most interesting predicted clusters producing potential valuable drugs from whole-genome sequences. We also note that a similar approach has been successful in bacterial genomes (30).

The immediate advantage of the FRIGG pipeline is the direct identification of clusters with a high likelihood of coding for useful bioactive compounds. Another major advantage is that the target of the compound, and, hence, the mode of action, is inherently known. Knowing the target saves a lot of time since linking the compound to the target is extremely difficult and time-consuming. Furthermore, several regular drug discovery steps can be eliminated, since possible uses of the compound are known from the beginning, allowing a direct route to testing for likely modes of action.

## RESULTS

### Pipeline development and input data.

As a starting point, we were interested in creating a pipeline for identifying clusters containing possible resistance genes from whole-genome sequencing data. In order to do this, we based the pipeline on the assumption that the resistance gene is found within a cluster and is a copy, a paralog, of an essential gene found elsewhere in the genome.

For testing, we used published complete and draft-quality fungal genome sequences. The input for the pipeline was chosen to be three different types of data derived from the genome sequence data: (i) predicted genes/proteins and functionally annotated proteins (for functional annotation, we used InterPro [19, 20]), (ii) predictions of secondary metabolite gene clusters (for this purpose, we used a reimplementation of the fungus-optimized SMURF algorithm [21], as described previously by Vesth et al. [6]), and (iii) groups of homologous protein sequences (we used a pipeline designed for *Aspergillus* data, creating homologous protein families based on single linkages of bidirectional BLASTp hits [as described in reference 6]). It is assumed that proteins with similar, although not necessarily identical, functions will be clustered into the same protein family. For our purpose, this is useful, as resistance and target genes will be grouped into one family.

As an extensive test data set, we used 51 *Penicillium* and *Aspergillus* species containing a total of 3,276 predicted clusters and 26,551 secondary metabolite genes.

**FIG 2:**
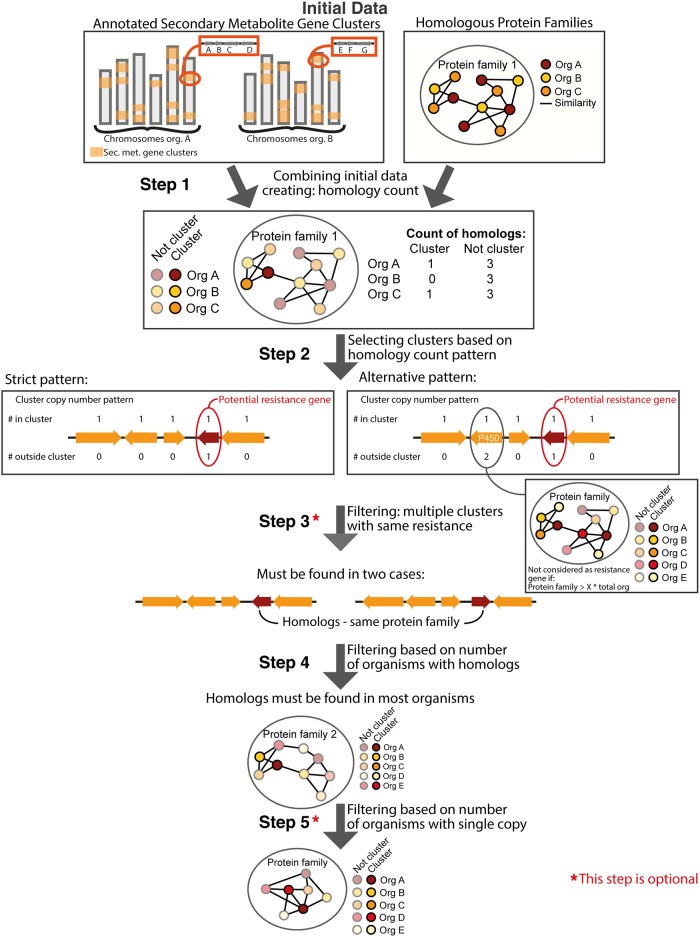
Overview of the pipeline illustrating the initial data of predicted secondary metabolite gene clusters and homologous protein families. In step 1, the initial data are combined to generate counts of how many homologs are found in each organisms and how many are within predicted clusters. In step 2, clusters are selected based on specific homology count patterns, either “strict,” where only one gene can have a homolog outside the cluster, or “alternative,” where genes belonging to large protein families are allowed. Step 3 is filtering of the selected clusters based on other clusters having a homologous resistance gene; this step is optional. In step 4, filtering based on the majority of the organisms should provide a homolog of the resistance/target genes. Step 5 is filtering based on the number of organism that should have only a single homolog.

In order to identify the most likely candidate clusters containing potential resistance genes, the pipeline includes a number of filtration steps (Fig. 2). Several of these are designed to deal with and/or minimize the effect of possible errors in assembly and annotation due to either inherent errors in sequencing technologies and predictions or errors caused by the assembly and sequence quality of draft genomes. Instead of attempting to correct these errors in the sequence or annotation, we attempt to mitigate their effect by creating filtering steps. Besides mitigating annotation and sequencing errors, the filtering steps are also implemented to deal with biological diversity and differences.

The strategy of the pipeline is thus to identify clusters containing resistance genes, assuming that the resistance genes are copies of essential genes. In this context, an essential gene is defined as a gene that has homologs in all the species included in the analysis.

### Steps 1 and 2: identification of the number of homologs for cluster genes.

The first step in the pipeline is to couple precomputed homolog protein families to the predicted cluster genes (Fig. 2, step 1). Following this, the number of homologs, the homology count, for each secondary metabolite gene in each organism/genome is identified. In addition, the numbers of homologs found in predicted clusters are recorded.

Next, clusters with potential resistance genes are selected based on a specific pattern of homology counts (Fig. 2, step 2). In this step, the user can select various levels of stringency for the selection pattern.

The most strict and simple selection pattern (Fig. 2, step 2, left) identifies clusters where only one of the genes has a homolog in the genome. It can have only one such homolog, and this homolog must not be part of another cluster. The gene with the homolog in the cluster is the presumed resistance gene, and the homolog outside the cluster is the presumed target gene. Using this selection criterion on our test data set (see Table S1 in the supplemental material, which includes GenBank accession numbers), 262 clusters were identified and divided into 141 potential resistance genes protein families (Table 1). This corresponds to 8% of the total clusters in the data set.

**TABLE 1:** Number of cases after each filtering step using various settings^*a*^

Step	No. of cases		
	Strict	Alternative (*X* = 2)	Alternative (*X* = 3)
2	141	255	202
3	45	88	67
4	19/14	38/25	32/22
4*	70/43	99/57	89/54
5	6/4	22/15	12/8
5*	31/16	59/33	41/22

a The columns represent three different cluster selection criteria at step 2, where “Strict” is the most conservative and “Alternative” (*X* = 2 and *X* = 3) is where the protein families are larger than 2 or 3 times the total number of organisms allowed in the cluster but not considered potential resistance genes. The rows represent each of the steps in the pipeline, and * indicates that step 3 was skipped. After step 4, the two numbers indicate that the resistance protein family has to have homologs in 90%/98% of the organisms in the data set in step 4.

10.1128/mSystems.00085-19.4 TABLE S1 Species used in this study, showing species name, section, and link to the JGI pages with the genomes. Download Table S1, CSV file, 0.00 MB. Copyright © 2019 Kjærbølling et al. 2019 Kjærbølling et al. This content is distributed under the terms of the Creative Commons Attribution 4.0 International license.

This “strict” selection pattern is very restrictive: if any of the other cluster genes have a homolog anywhere in the genome, the cluster will be filtered away. Many clusters contain tailoring enzymes with common functions, such as P450 or methyltransferases; these can be thought of as “household” functions in secondary metabolism. Clusters with common tailoring functions will frequently have several homologs, resulting in high homology counts, and the cluster will therefore fall out of the strict selection pattern, which may not be what the user wants. Another effect of the filtering is that the selected clusters often contain fewer genes (average size of 6.4 genes versus 8.2 genes for the total data set). The larger the cluster, the more likely it is that there are several genes with homologs in the genome. It is likely that clusters containing potential resistance genes are missed due to the strict selection criteria. Conversely, the share of bioactive clusters is increased after the selection.

### Alternative cluster selection pattern.

We wanted to create an alternative selection pattern to allow the presence of more tailoring enzymes and larger cluster sizes. As a method to do this, we added an analysis of the genes in the clusters. The most common InterPro domains (annotated in at least 1,000 cluster genes) were selected, the protein families belonging to each InterPro domain were identified, and the sizes of the protein families were noted. Here, we see that in our data set, the protein families belonging to each InterPro domain range in size from 1 to 475 proteins (Fig. S1).

10.1128/mSystems.00085-19.1 FIG S1 Common InterPro domains in secondary metabolism. Shown is visualization of the most common InterPro annotations of secondary metabolite genes (found in more than 1,000 secondary metabolite proteins) and the size of the protein families. Two horizontal lines indicate the recommended protein family size cutoffs where *X*_input_ is 2 (102 proteins) and 3 (153 proteins). Download FIG S1, PDF file, 0.2 MB. Copyright © 2019 Kjærbølling et al. 2019 Kjærbølling et al. This content is distributed under the terms of the Creative Commons Attribution 4.0 International license.

When protein families with several homologs in each organism appear in a cluster, the cluster will be discarded using the strict selection pattern. To avoid this, an “alternative” selection pattern was created to disregard large protein families as potential resistance genes and instead allow the proteins belonging to large protein families to have homologs in the organism (Fig. 2, step 2, right).

The size of the protein families will change depending on the data (e.g., the number of genomes included and how closely related the species are). The selection was therefore designed to be dependent on the number of organisms in the data set: if the protein family is larger than the total number of organisms in the data set multiplied by *X*, where *X* is a user input, then the gene is ignored as a potential resistance gene; e.g., if *X* is set to 2, the cutoff would be 2 times 5 organisms (Fig. 2, step 2). As the protein family illustrated has 11 members, it would be allowed in the pattern. If a value of 3 is selected, the cutoff would be 12, and the illustrated protein family is not large enough and this cluster would be eliminated.

In our test set, we used a value of 2 or 3 as the user input (*X*), thereby disregarding protein families with more than 102 or 153 members, respectively. With these selection criteria, 482 clusters (*X* = 2) and 388 clusters (*X* = 3) are identified, respectively, corresponding to 84% and 48% increases compared to the initial strict selection criteria (Table 1). Across these clusters, there are 255 and 202 different potential resistance gene families, corresponding to increases of 81% and 43%, respectively, showing that the strict measurement is indeed sensitive to large protein families.

### Step 3: filtering for resistance genes found in more than one cluster.

A filtering step is implemented in order to mitigate cluster prediction errors (Fig. 2, step 3). Cluster prediction algorithms are sensitive to the surrounding genes, but the inclusion of multiple genomes mitigates this problem. Therefore, we have included the option for FRIGG to return only clusters where at least one other identified cluster has a potential resistance gene belonging to the same protein family. Thereby, the members of the potential resistance gene protein family have to be found in at least two clusters, across multiple genomes.

Using these filtering criteria, 45, 88, and 67 of the previously identified potential resistance gene protein families are left for the strict pattern, the alternative pattern where *X* equals 2, and the alternative pattern where *X* equals 3, which correspond to 32 to 35% of the initial selected potential resistance gene cases (Table 1). As this is conservative, we have allowed for the user to skip this step.

### Step 4: filtering for clusters targeting essential proteins.

It is an underlying assumption that the target of the compound produced by the cluster should preferentially have an essential function, and thus, it should have protein members in all the species. By extension, the protein family in which the resistance proteins are found should therefore also be found in all genomes in the set. Here, we employ this definition of essential (present in all genomes) to allow for uncharacterized essential genes.

The addition of a filtering step to eliminate clusters where the resistance gene is not duplicating an essential protein family has two purposes: first, it makes it more likely that it is a resistance gene if there are homologs in all species, and second, it will be a more widely useful bioactive compound if the target is conserved in many species.

The implementation of the filtering step removes clusters where the putative resistance genes have homologs in fewer than 90 to 100% of the organisms (Fig. 2, step 4). The possibility of choosing values lower than 100% was implemented to allow for some data and prediction errors, such as incomplete genomes and imperfect gene annotations. Another reason for choosing a value lower than 100% is to allow some species to have a different mechanism and, thus, not the target gene.

We examined the effects of selecting 98% and 90% of the genomes (Table 1, step 4). Overall, the numbers of predicted resistance genes drop considerably, to fewer than one-half of those in step 3 with a 90% cutoff and fewer than one-third with a 98% cutoff.

As mentioned above, step 3 is conservative and optional. If step 3 is skipped and the essentiality filter is applied (Table 1, step 4*), fewer than half are retained for the 90% cutoff and around one-fourth are retained for the 98% cutoff.

### Step 5: filtering the number of organisms with single copies.

The final filtering step is related to the organisms in which the putative resistance genes have homologs. The assumption here is that the target gene should be a single essential gene and, therefore, that the majority of the species should have only a single copy.

This filtering step therefore removes protein families where more than 50% of the genomes have more than one homolog of the putative resistance gene (Fig. 2, step 5). This step is also optional. The effect of adding this selection criteria can be seen in Table 1 (steps 5 and 5*).

### Pipeline output.

The primary output of this pipeline is a list of protein families with potential resistance genes and fasta files containing all the proteins belonging to the identified family. The header of each entry includes the name of the organism, the section to which it belongs, the protein identification, the number of copies found in the organism, and whether it is in a selected cluster (StrictClust), a predicted cluster (Clust), or somewhere else in the genome (0), which is followed by the amino acid sequence.

If high experimental capacity is available, one option is to test all these cases; if the experimental capacity is limited, further analysis is needed to evaluate and select which cases to work with. In this example, with 51 species, we obtain 12 different outputs after step 5 using each of the various settings (Table 1). Starting from 3,276 clusters, we compare the outputs of all 12 settings and can combine them into 72 unique putative resistance gene families (Table S2). Four of these are found in every analysis, while 19 are found only once using a specific set of variables.

10.1128/mSystems.00085-19.5 TABLE S2 Overview of the 72 identified putative resistance gene families and the parameters with which they were identified. Each row represent a protein family, the columns represent the various settings, and “1” indicates that a protein in the protein family was identified as a potential resistance gene using these settings. Download Table S2, CSV file, 0.00 MB. Copyright © 2019 Kjærbølling et al. 2019 Kjærbølling et al. This content is distributed under the terms of the Creative Commons Attribution 4.0 International license.

With each filtering step, the number of clusters and putative resistance genes decreases, but the share of likely bioactive clusters and resistance genes increases.

### Computer-assisted curation for potential bioactive clusters.

Even with the above-described selection criteria, the pipeline still produces more candidate clusters than would be feasible for most academic laboratories to verify experimentally. Once the pipeline has been run and potential resistance gene cases have been identified, further analysis is needed to select the most promising candidates for experimental verification.

**FIG 3:**
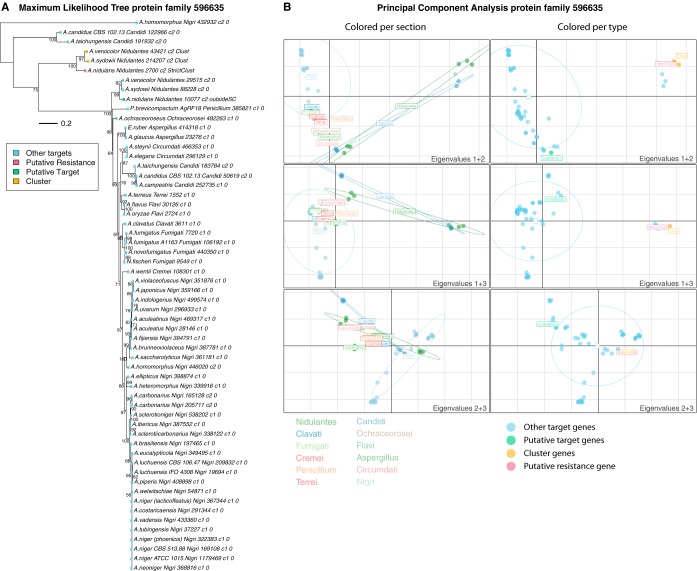
Phylogenetic tree and principal-component analysis of protein family 596635, including the fellutamide B resistance gene. (A) Phylogenetic tree of the protein family (596635) containing the fellutamide B resistance gene (protein identification 2700) from A. nidulans. The phylogenetic tree is a maximum likelihood tree and was created with 500 bootstraps. (B) Principal-component analysis of protein family 596635 containing the fellutamide B resistance gene (protein identification 2700) from A. nidulans. The panels to the left are colored based on the sections to which the proteins belong, while the panels to the right are colored based on if the protein is a putative resistance gene (StrictClust), in a predicted cluster (Clust), a target not in a cluster (0), and the homolog to the putative resistance gene (putative target) (outsideSC).

In order to do this efficiently, we use a combination of principal-component analysis (PCA), phylogenetic analysis, functional annotation, and comparison to the NCBI database using BLASTp in order to gain more knowledge about the potential cases (examples of the PCA and phylogenetic trees can be seen in Fig. 3 and in Fig. S2 and S3 in the supplemental material).

10.1128/mSystems.00085-19.2 FIG S2 Principal-component analysis of protein family 597268 containing two potential resistance genes (protein identifications 11595 and 32200) found in *A. oryzae* and A. flavus. The panels to the left are colored based on the sections to which the proteins belong, while the panels to the right are colored based on if the protein is found in a selected cluster (StrictClust) (green), not in a cluster (0) (blue), and the homolog of the ones found in selected clusters (outsideSC) (yellow). Download FIG S2, PDF file, 0.7 MB. Copyright © 2019 Kjærbølling et al. 2019 Kjærbølling et al. This content is distributed under the terms of the Creative Commons Attribution 4.0 International license.

10.1128/mSystems.00085-19.3 FIG S3 Phylogenetic tree of protein family 597268 containing two potential resistance genes (protein identifications 11595 and 32200) found in *A. oryzae* and A. flavus. The node labels show bootstrap values based on 500 bootstraps. The tip labels have the species name, the section, protein identification, the number of homologs in the species, and an indication of whether it is found in a selected cluster (StrictClust), in a predicted cluster (Clust), not in a cluster (0) and the homolog of the ones found in selected clusters (outsideSC). Download FIG S3, PDF file, 0.2 MB. Copyright © 2019 Kjærbølling et al. 2019 Kjærbølling et al. This content is distributed under the terms of the Creative Commons Attribution 4.0 International license.

The functional annotation and BLAST analysis are included to examine whether a potential resistance gene has a known function, if the function is essential, and if it would be a good drug target.

The PCA and phylogenetic trees are used to illustrate the evolutionary relationship between the proteins in the protein family. Resistance genes are expected to be a duplication of a target gene that is essential. Essential genes are under similar evolutionary pressures in all the species and are therefore closely related. The resistance gene, however, is under a different selection pressure, potentially expressed under a different subset of conditions, which is expected to be reflected in the sequence and, hence, the analysis, where resistance genes should form a separate group in the PCA or a separate clade in the tree compared to the target genes.

Another thing which can be considered is the synteny of the clusters, whether the order and functions of the genes are conserved when comparing clusters identified in two species. This can be used to evaluate cluster borders and similarity, but it requires at least two species to have similar clusters, which is not always the case.

### Examination of verified clusters.

As mentioned in the introduction, there are some known clusters in fungi containing a verified resistance gene using this mechanism. These include the mycophenolic acid cluster in P. brevicompactum and the fellutamide B cluster from A. nidulans. Both clusters were included in this study as validation controls to check if the known resistance genes would be identified using our pipeline.

The fellutamide B cluster is identified in all the outputs where step 3 was skipped, since this was the only cluster with this resistance gene and the selected homology count pattern. Thus, when filtering for replication of the pattern (step 3), it falls out of the analysis.

One hypothesis was that the potential resistance and target genes are exposed to different evolutionary pressures. This is expected to be reflected in the evolutionary pattern, where the essential target genes (under high pressure) will be closely related, whereas the resistance genes will show more variation. To investigate if this is the case, and to provide an example of the curation process, a phylogenetic tree and a PCA were performed for the protein family containing the fellutamide B resistance gene (protein family 596635) (Fig. 3). The PCA shows a clear distinction between the resistance genes found in clusters (Fig. 3B, red) and the other essential target genes (Fig. 3B, blue). The phylogenetic tree (Fig. 3A) shows two clear groupings: one large closely related group with all the target genes (blue) and one small group containing the resistance gene (red). The target genes are ordered in their phylogenetic groups, so species from the same section group together. The resistance gene from A. nidulans is grouped with a protein from Aspergillus sydowii and A. versicolor, both of which are found in clusters, indicating that they most likely also function as resistance genes and potentially can produce fellutamide B or a derivative or a different compound attacking the same target. A. versicolor is known to produce fellutamides C and F (22, 23), whereas A. sydowii, to our knowledge, has not been reported to produce derivatives of fellutamide. The clusters in A. versicolor and A. sydowii are similar to the A. nidulans fellutamide B cluster, with similar backbone enzymes (≥70%) and tailoring enzymes, but the A. versicolor and A. sydowii predicted clusters are larger, consisting of 19 genes, 7 more than in the A. nidulans cluster. Three of the extra genes have homologs in the genome and belong to small protein families, which is why these clusters are not identified in the first steps of the pipeline.

Near the resistance genes in the phylogenetic tree, there are also three other genes, found in Aspergillus homomorphus, A. taichungensis, and A. candidus, which are extra copies of the target genes, but they are not predicted to be in clusters. These could be cluster prediction errors. We therefore investigated whether the three species have a cluster similar to the A. nidulans fellutamide B cluster, but no backbone genes had a BLASTp hit identity of >37%, indicating that they most likely cannot produce any derivatives of fellutamide. Another explanation for the extra copy could be that it functions in fellutamide resistance, which could be useful if the species naturally grow near fellutamide-producing species.

The cluster of mycophenolic acid did not turn up in our outputs, so we investigated the cluster further to understand why. The predicted cluster containing the polyketide synthase (PKS) responsible for producing the core compound of mycophenolic acid consists of four genes: the PKS, a P450, a methyltransferase, and the resistance gene. The PKS and p450 are found in only one copy, and the resistance gene has one identified homolog, as expected. The methyltransferase, however, also has a homolog elsewhere in the genome. As the size of this protein family is only 23 members, it is not disregarded in the alternative pattern, and the cluster is filtered away in step 2. This shows that we lose some correct cases along the steps in the pipeline, which illustrates the importance of running the pipeline with multiple settings and inspecting the output carefully. The pipeline is sensitive to the number of organisms included, and the settings should be used while keeping this limitation in mind.

### Novel putative resistance gene.

In addition to the known clusters with resistance genes, several uncharacterized clusters were also identified. Here, we focus on one example where the potential resistance gene is found in clusters in *A. oryzae* (protein identification 11595) and A. flavus (protein identification 32200) (protein family 597268). The PCA showed a very clear picture of the resistance genes falling outside the group of target genes; the same is seen in the phylogenetic tree, where the target genes also follow the expected phylogeny, with species from the same section grouping together (Fig. S2 and S3). Besides the identified putative resistance genes in Aspergillus oryzae and A. flavus, several other species (Aspergillus wentii, A. piperis, A. candidus, A. taichungensis, A. campestris, A. novofumigatus, and *P. brevicompactum*) have an additional gene, but these are not found in predicted clusters, suggesting that they have the resistance but not the biosynthesis.

The predicted clusters in A. oryzae and A. flavus both consist of four genes: an acetyltransferase (InterPro accession numbers IPR000182 and IPR016181), the predicted resistance gene, a nonribosomal peptide synthetase-like synthetase, and a gene belonging to the major facilitator superfamily (InterPro accession number IPR011701). The putative resistance gene has annotations involved in “signal transduction response regulator, receiver region,” and “signal transduction histidine kinase, core” (InterPro accession numbers IPR001789 and IPR005467). Using BLASTp to investigate the function of the putative resistance gene, the top hits have functions such as “two-component osmosensing histidine kinase (Bos1)” (GenBank accession number RAQ55620.1). Based on this information, it seems likely that this is a cluster containing a previously unidentified resistance gene where the compound inhibits a conserved histidine kinase. In order to fully determine this, this will have to be experimentally validated in the future.

## DISCUSSION

The pipeline was designed to identify secondary metabolite gene clusters (clusters) containing potential resistance genes. It is a delicate balance of filtering away as many clusters as possible to narrow down the field to the best candidates while keeping as many clusters with potential resistance genes as possible.

We have chosen an approach where we make no assumptions about the function or makeup of resistance genes besides being homologs of a gene shared by most organisms in the data set (a mock measure for essentiality). Another approach could be to screen for specific classes of essential genes or resistance targets within predicted clusters. This approach was used in another study where 86 bacterial *Salinispora* genomes were mined for duplicated genes involved in central metabolism colocalizing with clusters. Clusters containing putative fatty acid synthase resistance genes were identified, and these were shown to be involved in the biosynthesis of thiotetronic acid natural products, including thiolactomycin, which is a well-known fatty acid synthase inhibitor (24). This approach builds on knowledge of housekeeping genes, thus requiring extensive knowledge of the primary metabolism. In filamentous fungi, there are still many uncharacterized genes; therefore, there is a risk of missing interesting resistance genes using that approach. To avoid this, we chose to use a wider approach based only on the homology copy number pattern of predicted secondary metabolism genes and not on the functions. The underlying assumption is that our pipeline identifies conserved household genes with a homolog in a cluster. Using our approach, we avoid limiting the search space to only known mechanisms, thereby making it possible to find new essential mechanisms and drug targets. Our non-function-impelled approach can be used on organisms with both little and extensive available knowledge. Finally, the setup makes it possible to search the identified clusters afterwards with a criterion such as the presence of primary metabolism genes. The pipeline has been designed for a specific data setup, but it is equally possible to apply the approach to other data sets.

In applying the pipeline, it is important to select the input data carefully. Genomes from very distantly related species might not give meaningful homologous protein families using our cutoffs, since proteins with similar functions might be more different than our cutoffs allow. Step 1 in the pipeline combines the input data and creates a table of homology counts for all cluster genes in the input set. In this step, no filtering or selection is done, and hence, there are no parameters to tweak.

In step 2, strict or alternative patterns for the cluster homology counts can be selected. Using the strict selection criteria, only 8% of the clusters meet the criteria. When using selection patterns that are too restrictive, there is a risk of creating false-negative results, thus filtering away good cases. As mentioned above, many common secondary metabolite genes are likely to have homologs and belong to large protein families. Of the cluster genes belonging to this test data set, 12% and 6% belong to protein families larger than 102 and 153 proteins, respectively, and notably, these are found in 51% and 37% of the total clusters. Thus, using the strict selection pattern, up to one-half of the clusters are likely to be discarded due to the homology count pattern of standard cluster genes, which is highly conservative and can cause false-negative results. We therefore recommend the use of the alternative selection pattern. If the species in the data set are distantly related, the size of the protein families might be smaller, and therefore, a lower *X*_input_ is recommended. The lowest value of *X*_input_ that we recommend is 1.5; when using a lower value, the risk of disregarding true resistance gene families becomes very high.

Step 3, filtering for more than one cluster having the putative resistance gene, was designed to avoid false-positive results due to cluster prediction errors. This step is useful for data that include closely related species likely to have similar clusters. If the data consist mainly of distantly related species, it is less likely that similar clusters will be found in more species, and hence, the risk of filtering away good cases increases, thereby making false-negative results. In the test data, about two-thirds of the cases are filtered away in this step. If running the pipeline and more than two-thirds are filtered away, we suggest skipping this step. With distantly related species, we suggest always skipping this step.

Step 4 was made to avoid false-positive results, by checking that the resistance gene is a homolog of an essential gene. One can set the percentage of species required to have the homolog. If the species are distantly related, it is more likely that some species have a different essential household mechanism and hence do not have a copy of the household/target gene. A lower percentage of organisms that should have a homolog is thus recommended in this case. Another reason for choosing a lower percentage is when the quality of the genome sequence data is low, with incomplete genomes, or when the data include novel species distantly related to model organisms, where the gene prediction algorithms might not work as efficiently. In these cases, absent homologs could be false-negative results and should thus be allowed for.

Step 5 was also added to avoid false-positive results; in this step, at least 50% of the species should have only a single homolog. This filtering step is independent of the quality of the data and the relatedness of the species, and we therefore recommend using this at all times. Here, 30 to 60% of the cases are left after this filtering.

In the future, it could be interesting to investigate the transcription of target and resistance genes in order to investigate if there are differences in expression patterns and if these can be applied in the search.

### Conclusions.

In this study, we have created a method to identify clusters responsible for producing bioactive compounds. The approach that we have developed is based on a specific resistance mechanism and paves the way for rational selection of promising bioactive clusters from whole-genome data. The FRIGG pipeline was designed for relatively closely related fungal genomes; however, several filtering steps and parameters can be tweaked to fit different kinds of data and to deal with the most likely errors from predictions and draft genomes.

We have tested the developed pipeline on 51 *Aspergillus* and *Penicillium* genomes, using various settings. By applying these settings, we identified 72 unique putative resistance genes and clusters in total. In addition, the previously characterized fellutamide B resistance gene *inpE* was successfully identified with this pipeline, confirming the accuracy and applicability of the pipeline to such cases of resistance mechanisms.

As more and more genomes are sequenced, the relevance of this approach will increase, and it will become a useful method for selecting which clusters to focus on in the hunt for novel drugs such as antifungals, anticancer drugs, and antimicrobial compounds.

## MATERIALS AND METHODS

### Fungal species.

The data consisted of 50 *Aspergillus* and 1 *Penicillium* species with available whole-genome sequencing data, which were downloaded from the JGI database (5, 6, 25). Species information, including GenBank accession numbers, can be found in Table S1 in the supplemental material.

### Input data.

All data are stored as csv/sql files in a folder on Zenodo and can be found at https://doi.org/10.5281/zenodo.2560245. The data used in the pipeline were organized in a MySQL database; an overview of the input data can be found in the file Input_data_pipeline.txt. The data include predicted secondary metabolite gene clusters based on an implementation of the SMURF pipeline (21) described previously (6). Homologous protein families were created with *Aspergillus*-optimized parameters based on single linkage of bidirectional BLASTp hits with an identity of ≥50% and a sum of query and hit coverage of ≥130%, as described previously (6). InterPro annotations of the proteins (19, 20) and gff information were also used.

### Pipeline.

All scripts and additional information files are available at Zenodo (https://doi.org/10.5281/zenodo.2560245). The pipeline was created using Python, and the investigation of specific protein families was conducted in R (26). The versions and packages used can be seen in version information files. For alignment of sequences of the protein families, clustalo (27) was used, and sequences were trimmed with Gblocks (28, 29) using a Python script, also included in the folder. A readme file describes the various steps in the pipeline, including the inputs, outputs, how to run them, and which parameters can be changed. The first step of the pipeline is the most time-consuming, combining the protein families and the SMURF data to create “homology count” data, which takes approximately 7 h on a standard desktop computer for the data used here. The following selection and filtering steps take 8 to 40 min, depending on the parameters used.
