# Supplementary figures and images for: Resistance Gene-Directed Genome Mining of 50 Aspergillus Species
Source: mSystems. 2019 May 14;4(4):e00085-19. doi: 10.1128/mSystems.00085-19 (PMC6517689; doi:10.1128/mSystems.00085-19)

Size of hfam by IPR

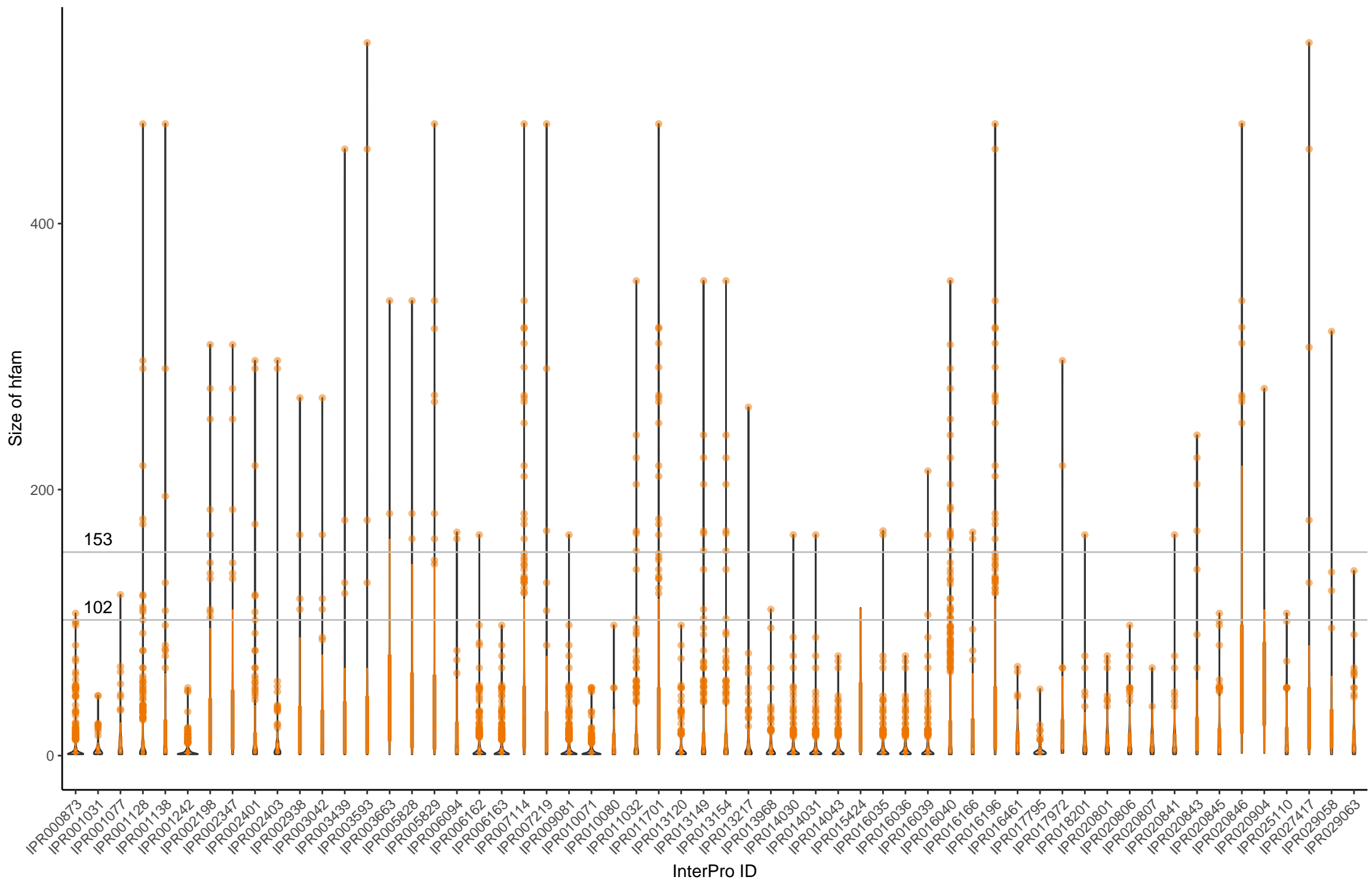

Supplement: FIG S1 [file mSystems.00085-19-sf001.pdf]

# PCA- protein family 597268

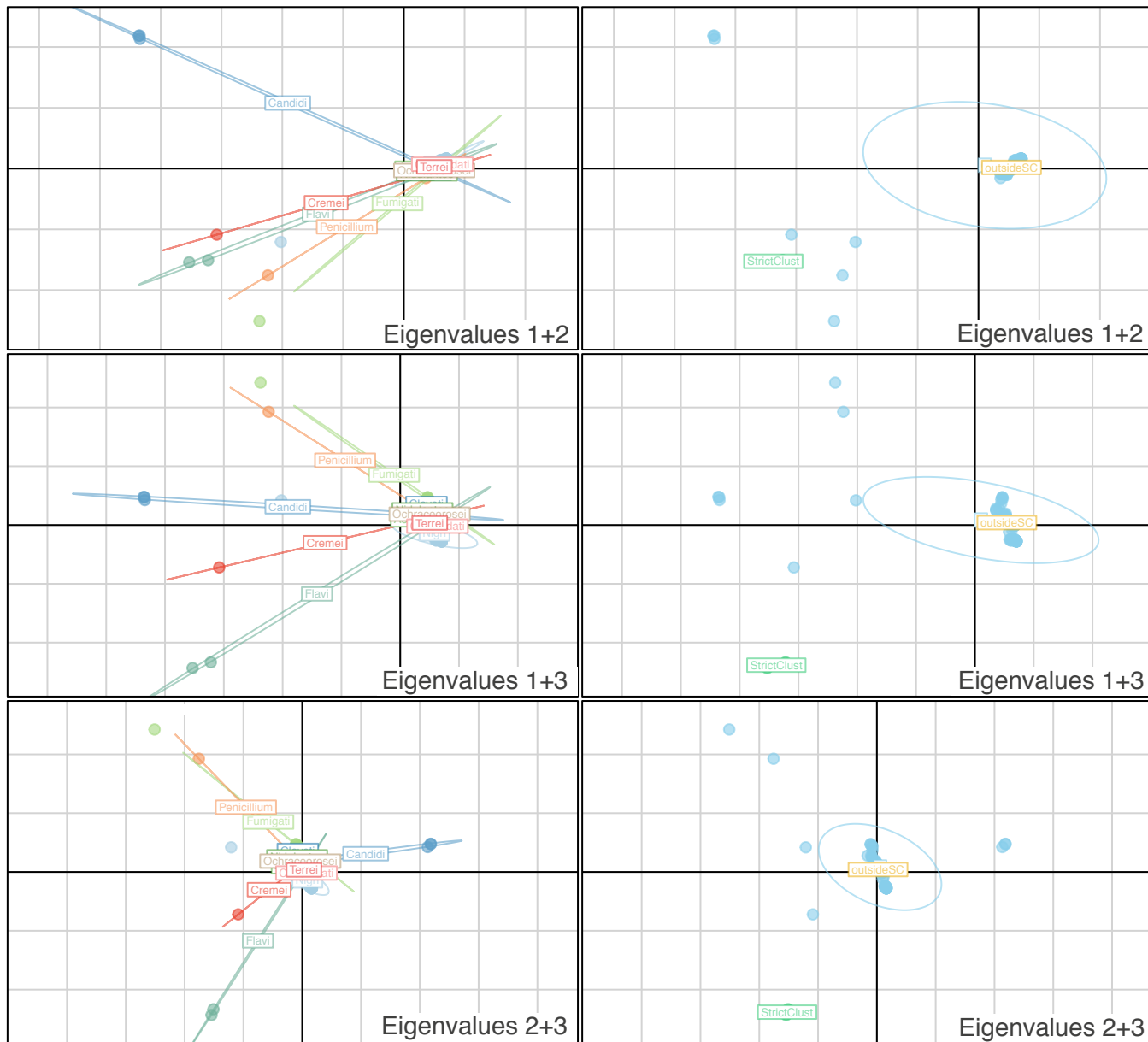

Supplement: FIG S2 [file mSystems.00085-19-sf002.pdf]

Maximum Likelihood Tree Hfam 597268 clust flag

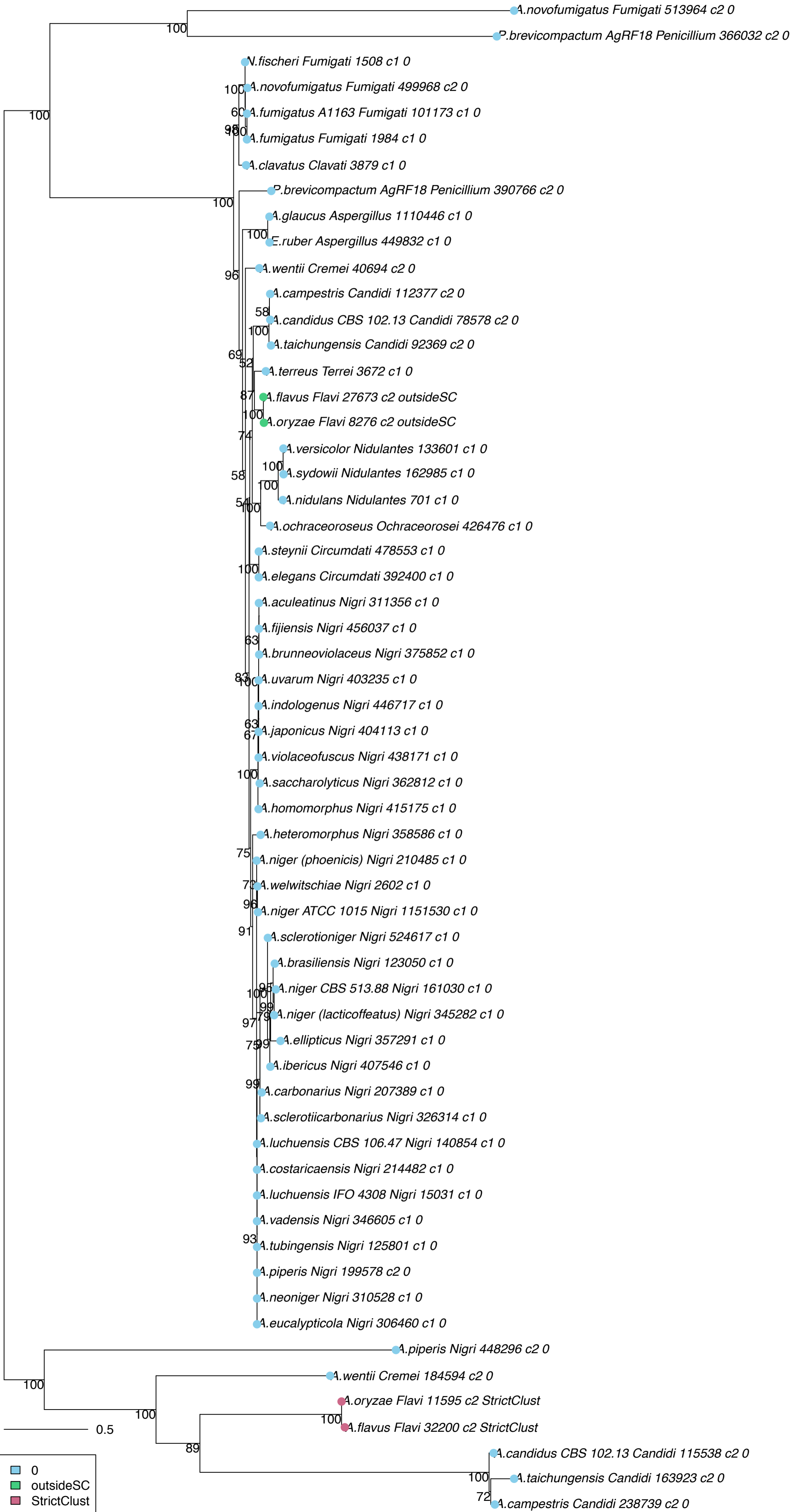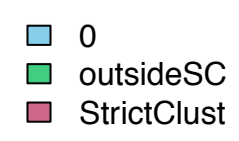

Supplement: FIG S3 [file mSystems.00085-19-sf003.pdf]
